# Characterizing the diversity of active bacteria in soil by comprehensive stable isotope probing of DNA and RNA with H_2_^18^O

**DOI:** 10.1002/mbo3.230

**Published:** 2015-02-04

**Authors:** Elizabeth A Rettedal, Volker S Brözel

**Affiliations:** 1 Department of Biology and Microbiology, South Dakota State UniversityBrookings, South Dakota, 57007; 2 Department of Microbiology and Plant Pathology, University of PretoriaPretoria, 0004, South Africa

**Keywords:** Bacterial diversity, DNA, H_2_^18^O, RNA, SIP, soil

## Abstract

Current limitations in culture-based methods have lead to a reliance on culture-independent approaches, based principally on the comparative analysis of primary semantides such as ribosomal gene sequences. DNA can be remarkably stable in some environments, so its presence does not indicate live bacteria, but extracted ribosomal RNA (rRNA) has previously been viewed as an indicator of active cells. Stable isotope probing (SIP) involves the incorporation of heavy isotopes into newly synthesized nucleic acids, and can be used to separate newly synthesized from existing DNA or rRNA. H_2_^18^O is currently the only potential universal bacterial substrate suitable for SIP of entire bacterial communities. The aim of our work was to compare soil bacterial community composition as revealed by total versus SIP-labeled DNA and rRNA. Soil was supplemented with H_2_^18^O and after 38 days the DNA and RNA were co-extracted. Heavy nucleic acids were separated out by CsCl and CsTFA density centrifugation. The 16S rRNA gene pools were characterized by DGGE and pyrosequencing, and the sequence results analyzed using mothur. The majority of DNA (∽60%) and RNA (∽75%) from the microcosms incubated with H_2_^18^O were labeled by the isotope. The analysis indicated that total and active members of the same type of nucleic acid represented similar community structures, which suggested that most dominant OTUs in the total nucleic acid extracts contained active members. It also supported that H_2_^18^O was an effective universal label for SIP for both DNA and RNA. DNA and RNA-derived diversity was dissimilar. RNA from this soil more comprehensively recovered bacterial richness than DNA because the most abundant OTUs were less numerous in RNA than DNA-derived community data, and dominant OTU pools didn't mask rare OTUs as much in RNA.

## Introduction

Soils constitute a critical ecosystem rich in microbial diversity (Brussaard 1997), and may contain a billion or more bacterial cells per gram (Torsvik et al. 1990; Whitford 1996). Soil bacterial diversity is generally high, indicating a complex microbial habitat (Elshahed et al. 2008; Youssef and Elshahed 2009; Will et al. 2010), but its recalcitrance to cultivation attempts has lead to widely accepted culture-independent approaches (Ward et al. 1990; Hugenholtz et al. 1998; Sharma et al. 2005). Culture-independent analyses are most frequently based on nucleic acid semantides (Zuckerkandl and Pauling 1965) extracted from soil samples, and as such the results reflect the diversity of the nucleic acid present. The 16S rRNA gene is often the target of community analysis because it currently represents the most universally usable target for sequence analysis (Clarridge 2004; Pace 2009). Extracted nucleic acids derive from unknown proportions of active, dormant, dead and lysed cells, and free DNA. Extracellular DNA can persist in the environment, and dormant organisms may not possess any significant function within the bacterial community (Holm-Hansen et al. 1968; Lorenz and Wackernagel 1987; England et al. 2004). Any downstream analyses of these DNA extracts will reflect the diversity in the sample extract, but not the composition of the active or living subset. Some researchers have used ribosomal RNA (rRNA) in place of DNA, arguing that RNA is less stable, and that these analyses would therefore yield a reflection of active bacterial diversity (Wagner 1994; Felske et al. 1998). Active bacteria reflect a range of states, such as growth and metabolic activity (Bremer 1996). Cellular growth entails a series of polymerizing processes including envelope synthesis, replication, ribosomal synthesis, and cell division. Nondividing cells may still be metabolically active, both through biochemical activity and protein and ribosomal remodeling.

Stable isotope probing (SIP) entails the incorporation of heavy isotopes such as ^13^C or ^15^N into the nucleic acids of bacteria that metabolize the isotopically labeled substrate. Bacteria able to utilize the substrate will incorporate some of the heavy isotope into their DNA and RNA. This labeled nucleic acid can be separated from the unlabeled by density gradient centrifugation and used for culture-independent analyses. While SIP studies have focused on particular groups such as methanotrophs (Morris et al. 2002; Liu and Conrad 2010; Chen 2011), the introduction of H_2_^18^O as a labeled substrate has widened the potential of this technique (Schwartz 2007; Angel and Conrad 2013). All growing cells need to uptake water, so amending samples with H_2_^18^O should allow labeling of active microbes and recovery of the their nucleic acids (Schwartz 2007, 2011). Recent evidence indicates that heavy oxygen in H_2_^18^O is incorporated across structural components of DNA, and that the heavy isotope does not exchange with water once the DNA has been extracted (Aanderud and Lennon 2011).

DNA-SIP reflects DNA replicating after addition of the label, yielding an inventory of all dividing cells, while RNA-SIP reflects transcriptionally active cells producing new ribosomes (Chen 2011; Manefield and Whiteley 2011). Several studies have compared community composition based on DNA versus RNA (Duineveld et al. 2001; Nogales et al. 2001; Moeseneder et al. 2005) and DNA versus RNA-SIP (Bernard et al. 2007; Liu and Conrad 2010; Dumont et al. 2011). Here, we report on a bacterial community analysis of agricultural soil based on total versus SIP-labeled DNA and rRNA, using H_2_^18^O. The term “active” will be used to refer to de novo synthesis of the DNA or RNA. Isotopically labeled heavy DNA (HDNA) reflects cells active in replication while heavy RNA (HRNA) represents cells with newly synthesized ribosomes.

## Methods

### Soil collection and labeling

All experiments were performed on Brandt silty clay loam soil consisting of 150 g/kg sand, 560 g/kg silt, 270 g/kg clay, with an organic matter content of 2.7% and a pH of 7.2, obtained from the Aurora Research Farm (Brookings, SD). The field had a history of rotation between various crops, last cultivated with soybean (*Glycine max*). A 30 × 30 cm area of dry soil was collected to a depth of 10 cm. The top soil (10 cm) was homogenized by shaking vigorously for 5 min. For soil microcosms, 10 g soil of the homogenized soil was distributed into each of four sterile conical tubes (50 mL). Two soil microcosms were supplemented with 1 mL heavy isotope water (Cambridge Isotope Laboratory, 97% labeled ^18^O), and two with 1 mL sterile dH_2_O and processed as detailed below (Fig. S1). To ensure even distribution of water and complete wetting of soil, the conical tubes were mixed by vortexing at maximum speed for 5 min. Samples were incubated at ambient temperature for 38 days to allow for incorporation of label by slower growing bacteria, and then stored at −20°C until nucleic acids could be extracted.

### Nucleic acid extractions from soil

The RNA Powersoil Total RNA Isolation Kit and DNA Elution Accessory Kit (MoBio, Carlsbad, CA, USA) were used to simultaneously extract the DNA and RNA from 2 g of soil from each of the ^18^O-labeled (2 tubes) and unlabeled tubes (2 tubes). DNA and RNA from each extract was quantified fluorometrically using the HS dsDNA and RNA Assay kits (Invitrogen, Carlsbad, CA, USA), with a Qubit fluorometer.

### Stable isotope probing

#### CsCl SIP

The DNA-SIP separation protocol was modified from Schwartz (2007). DNA from the duplicate labeled and unlabeled microcosms was separated in 10.4 mL polycarbonate tubes (Beckman Coulter, Brea, CA, USA) in a CsCl gradient consisting of 7 mL of saturated CsCl, 3.1 mL of gradient buffer (Schwartz 2007), 15 *μ*L of ethidium bromide (10 mg/mL), and 1000 ng of DNA from each of the duplicate microcosms (duplicates combined into a single tube). Separation was performed using a MLA-55 rotor in an ultracentrifuge (Beckman Coulter) at 201,800*g* for 72 h at 18°C. The labeled and unlabeled tubes were run simultaneously to ensure identical gradient centrifugation conditions. DNA was visualized with UV illumination to confirm separation into bands. Points above and below the visualized separation were marked on the tubes to indicate which sections of the gradient tube contained the separated bands. The gradients were fractioned into 100 *μ*L aliquots from the top down. Forty fractions in total were recovered into tubes. Six gradient fractions of 1 mL were recovered below the lowest marked sections. The DNA was recovered using ethanol precipitation (Sambrook and Russell 2006) and resuspended in 30 *μ*L sterile dH_2_O. The 1 mL fractions were confirmed to have no detectable DNA (Qubit HS DNA).

As a secondary control to confirm the separation and repeatability of the CsCl density centrifugation, we cultivated *Bacillus subtilis* 168 in labeled or regular water-based LB and performed the above centrifugation protocol and visualization on the extracted DNA samples.

#### CsTFA SIP

The RNA-SIP separation protocol was modified as previously described (Whiteley et al. 2007). RNA from duplicate labeled and unlabeled microcosms was separated in 2 mL polyallomer tubes (Beckman Coulter) in a CsTFA gradient consisting of 1.755 mL CsTFA (GE Healthcare Life Sciences, Pittsburgh, PA, USA), 72 *μ*L deionized formamide, 287 *μ*L gradient buffer (Schwartz 2007), and 750 ng of RNA from each of the duplicate microcosms (duplicates combined into a single tube). Separation was performed using a TLA-120.2 rotor in an ultracentrifuge (Beckman Coulter) at 89,000*g* for 50 h at 18°C. The labeled and unlabeled tubes were run simultaneously to ensure identical gradient centrifugation conditions. The gradients were aliquoted into twenty 100 *μ*L fractions from the top down. RNA was precipitated as described above for DNA. Samples were resuspended in 40 *μ*L of sterile dH_2_O and placed at 60°C for 10 min to aid in dissolving the pellet.

Absence of DNA in RNA was confirmed by PCR performed as described below. rRNA was reverse-transcribed to single-stranded cDNA using primer R907 (Teske et al. 1996) and M-MLV Reverse Transcriptase (Promega, Madison, WI, USA) by following the manufacturer's protocol.

### Individual fraction DGGE

**Figure 1:**
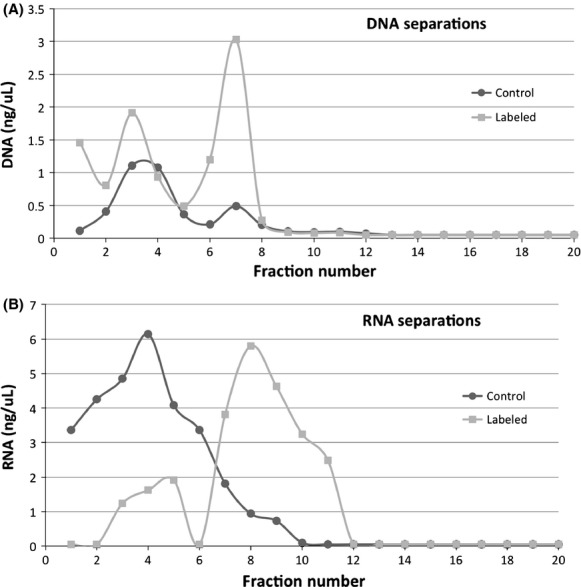
DNA (A) and RNA (B) concentrations in fractions taken from single CsCl and CsTFA density gradient separations performed on labeled and unlabeled (control) samples. Fraction numbers reflect samples taken from top to bottom. (Only 20 fractions from the CsCl gradient are visualized here (remaining 20 were not detectable by Qubit quantifications)). The light and heavy bands for the DNA are represented by fractions 3–4 and 6–7 and 3–5 and 7–11 for the RNA.

Multiple fractions representing light and heavy peaks were recovered from the gradient tubes. Concerns that SIP centrifugations may separate bacterial community members differently along a density gradient (Buckley et al. 2007) led us to compare multiple fractions representing heavy or light bands (Fig.1) by PCR-DGGE (Denaturing Gradient Gel Electrophoresis). Heavy or light fractions containing either DNA (fractions 3–4 and 6–7) or cDNA (fractions 3–5 and 7–11) were PCR-amplified targeting the V3-5 region of the 16S rRNA gene for DGGE using primers F357-GC (Muyzer et al. 1993) and R907 (Teske et al. 1996). PCR was performed using a 25 *μ*L reaction mixture containing 0.75 U GoTaq polymerase (Promega), 1X PCR buffer, 1.5 mmol/L MgCl_2_, 0.4 mg/mL bovine serum albumin, 4 *μ*mol/L each primer, 200 *μ*mol/L dNTPs, and 10 ng template DNA or cDNA. Thermocycling conditions were as follows: initial denaturation at 94°C for 5 min, followed by 35 cycles of 94°C for 30 sec, 55°C for 45 sec, and 72°C for 1 min, and a final elongation at 72°C for 7 min. PCR products (200 ng) were resolved on a 35–65% DGGE gradient gel and the gel images captured and analyzed as previously described (Rettedal et al. 2010). DGGE banding patterns were visually similar and clustered together (Euclidean paired group analysis) for the multiple fractions representing a heavy or light band from a particular semantide (e.g., all HDNA fractions cluster together, all light DNA fraction cluster together, etc.). This suggested that multiple fractions represented similar bacterial diversity and community members were not separated differently along the gradient.

### Pyrosequencing

Nucleic acid extractions from each microcosm were pooled in equal volumes to create a single template for each of the different samples. The template types prepared for 454 sequencing were unseparated (total) DNA extract from labeled soil (TDNA), labeled (heavy) DNA (HDNA), unseparated (total) RNA from labeled soil (TRNA), and labeled (heavy) RNA (HRNA) (Fig. S1). The labeled samples were comprised of equal volumes of multiple fractions (Fig.1) representing the separated heavy band. The V3-5 region of 16S rRNA gene pool was amplified using high-fidelity PCR master mix (Roche, Madison, WI, USA) in triplicate 20 *μ*L reactions using 2 ng DNA or cDNA as template, and F357 (Muyzer et al. 1993) and R907 primers with required Roche pyrosequencing tags and sorting tags attached (Roche 2009) (Table S1). The number of PCR cycles used was optimized for each sample to generate the minimum amount of amplification required for 5 *μ*L to produce a faint band on a gel. This has been shown to decrease PCR bias by reducing the over-amplification from certain genomes due to their 16S rRNA variable copy number, and decrease PCR artifacts and chimera generation (Farrelly et al. 1995; Polz and Cavanaugh 1998; Qiu et al. 2001). The triplicate PCR products were pooled and purified using a Gel Extraction kit (Qiagen, Valencia, CA, USA). About 40 ng of PCR product from each sample were combined for pyrosequencing on a Roche 454 Titanium GS-FLX sequencer at the University of Illinois Biotechnology Center utilizing 1/4th of a total plate.

### Sequence processing and analysis

Sequence quality control, processing, and analysis were performed using mothur following the SOP of Schloss et al. (2009, 2011). Following initial processing, sequences occurring only once among the entire data set were removed to reduce noise in sample comparisons. All samples were randomly subsampled to 5167 sequences to equalize the reads between samples. Sequences were binned at 3% difference for all analysis. Mothur was used to calculate the Shannon and Simpson diversity indices, Good's coverage, and rarefaction curves of the individual samples. The distance matrices between samples were calculated using both the Morisita-Horn and Jaccard indices in mothur. MEGAN was used to construct taxonomic graphs at various levels of classification (Mitra et al. 2011) and R was used to construct a heatmap of OTU (Operational Taxonomic Unit) abundance utilizing the gplots package (Team 2010; Warnes et al. 2013).

### Community analysis DGGE

In order to conduct an alternative analysis to pyrosequencing, PCR-DGGE was performed. DNA from soil from a nearby field plot subject to different agronomic history was used as out-group. The PCR was performed using replicate 25 *μ*L reactions and 2 ng of template (as described above) and DGGE was performed as above, but using a 35–60% gradient and loading 150 ng of DNA per lane.

## Results

### SIP and gradient centrifugation

The CsCl DNA gradient revealed two distinct bands from labeled samples, and one band from the unlabeled control samples. DNA from *B. subtilis* 168 cultured in either heavy or regular water-based LB broth served as secondary controls to verify repeatability of gradient centrifugation methodology. We did not observe three bands in our labeled gradient as previously reported for H_2_^18^O-DNA SIP by Schwartz (Schwartz 2007). The higher organic content of the agricultural soil in our experiment (2.7% vs. 1.18% (Schwartz 2007)), combined with longer incubation time may have supported more growth, with more effective incorporation of ^18^O. CsTFA gradients of RNA from labeled samples yielded a small “light” and larger “heavy” peak, while a single peak was observed for the unlabeled control (Fig.1), validating the success of the separation.

### Pyrosequencing quality control and experimental conditions

Following quality control and processing 22,425 sequences of an average length of 138 bp remained. To equalize sequence reads between samples all samples were subsampled to 5,167 sequences. The experimental protocols and conditions used for this study were tightly controlled during all steps in order to make semi-quantitative comparisons between the nucleic acid samples possible. This included amplification using equal quantities of DNA or cDNA, running equal numbers of replicates, and optimizing PCR conditions.

### OTUs, diversity measurements, and sample coverage

The total number of OTUs and Shannon and Simpson diversity indices were determined at 3% difference (Table1), sufficient to absorb errors in pyrosequencing (Acosta-Martínez et al. 2008; Kunin et al. 2010; Lim et al. 2010). The HRNA contained the greatest number of OTUs, followed by TRNA, HDNA, and TDNA. Both the Shannon and Simpson diversity indices indicated the same trends as the OTU counts, however, the difference in diversity between the two RNA samples was less than the DNA samples (Table1).

**Table 1:** The number of sequences and OTUs, Shannon and Simpson diversity indices, and Good's coverage as determined for each sample in the study

Sample	No. of sequences	No. of OTUs	Shannon index	Simpson index	Good's coverage
TDNA	5,167	635	5.09	0.0165	0.955
HDNA	5,167	689	5.33	0.0134	0.957
TRNA	5,167	734	5.64	0.0076	0.961
HRNA	5,167	776	5.69	0.0074	0.955

T(xxx) represents the total nucleic acids and H(xxx) represents the labeled nucleic acids.

To determine how well the soil bacterial community was sampled, rarefaction curves and the Good's coverage were determined. The Good's coverage (Esty 1986) estimates were around 96% for all samples suggesting that the dominant taxa had been recovered but some of the more rare OTUs had not (Table1). The rarefaction curves showed that the nucleic acid samples did not approach asymptote, suggesting a greater sequencing effort was required to recover the present richness (Fig. S2). Rarefaction curves also provide an alternative measure of the relative diversity (richness) between samples and have the additional advantage of not being influenced by the number of sequences. It indicated that the HRNA group had the greatest richness, followed by TRNA, HDNA, and TDNA.

### Sample comparisons

The Morisita-Horn index calculates the community structure dissimilarity between two samples. This measure was chosen because it considers sequence abundance and is not affected by sample number and species richness (Wolda 1981, 1983). The Morisita-Horn indices indicated that the TDNA–HDNA and TRNA–HRNA community structures were very similar (≥90%) while DNA and RNA communities only retained around 50% similarity (Table2). This suggested that the predominant OTUs within each nucleic acid type had active representatives of similar proportions. It also indicated that the DNA and RNA extracts reflected structurally dissimilar communities. The Jaccard index was also determined to highlight the contrast between shared OTUs and overall community structure dissimilarity. Despite the Jaccard index (shared OTUs) values hovering between 40% and 50% (Table2), the unshared OTUs made up a much lower percentage of the total sequences (less than 20%) indicating many were of low abundance (Table3). Among samples of the same nucleic acid type these unshared OTUs made up less than 10% of the total sequences (Table3).

**Table 2:** The calculated Morisita-Horn indices (shared community structure) and Jaccard indices (shared OTUs) for paired sample comparisons (e.g., TDNA/HDNA compares the dissimilarity between the total DNA and heavy (labeled) DNA sample)

Comparison	Morsita-Horn	Jaccard
TDNA/HDNA	**0.059**	0.416
TDNA/TRNA	0.445	0.510
TDNA/HRNA	0.550	0.512
TRNA/HDNA	0.435	0.497
HDNA/HRNA	0.529	0.469
TRNA/HRNA	**0.104**	0.407

A lower number indicates a greater similarity between samples. T(xxx) represents the total nucleic acids and H(xxx) represents the labeled nucleic acids. The bolded numbers highlight highly similar community structures.

**Table 3:** The calculated percentage of shared sequences is the fraction of sequences that belong to shared OTUs between sample pairs

TDNA	HDNA	TRNA	HRNA	
	0.9245	0.8182	0.8337	TDNA
0.9498		0.8802	0.8724	HDNA
0.9264	0.911		0.911	TRNA
0.92	0.9065	0.9332		HRNA

The displayed percentage is the fraction of sequences that the sample on the horizontal axis shares with the sample on the vertical axis. T(xxx) represents the total nucleic acids and H(xxx) represents the labeled nucleic acids

### RDP library classification comparison

**Figure 2:**
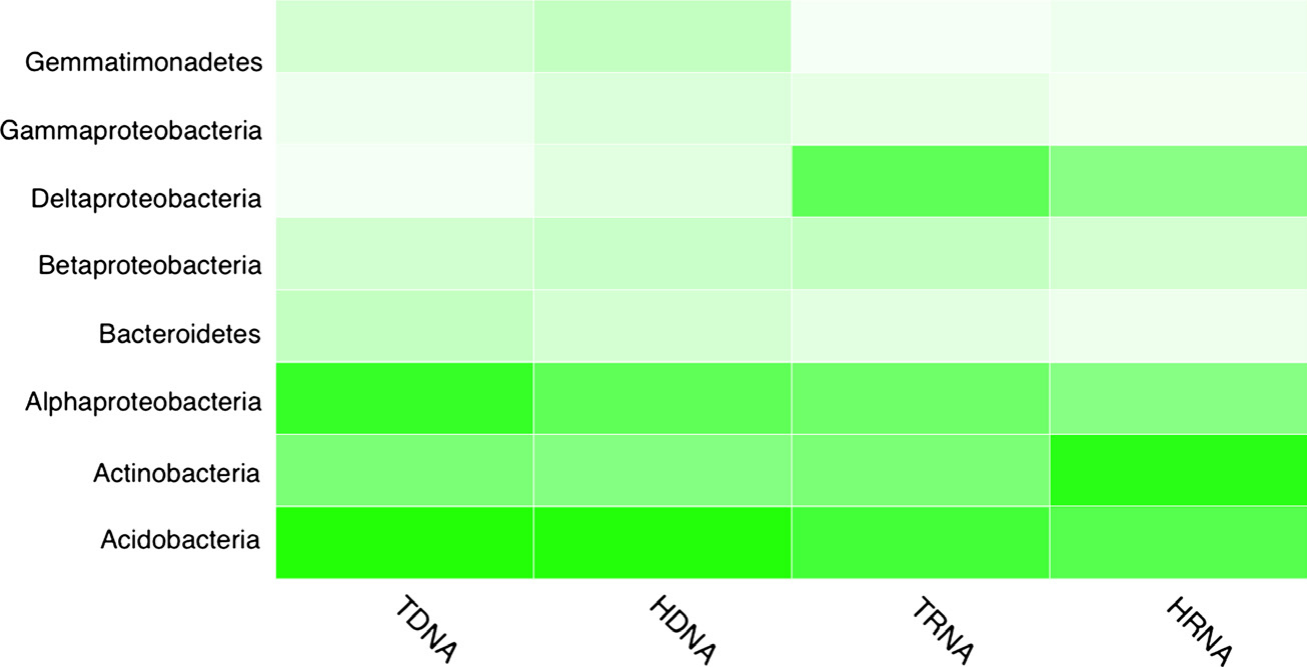
A MEGAN-generated heatmap that displays the taxonomic abundance of the samples at a class level classification. T(xxx) represents the total nucleic acids and H(xxx) represents the labeled nucleic acids.

Using output from mothur's taxonomic classifications and sequence abundance data, MEGAN (Huson et al. 2007) was used to construct heatmaps of class, order, and family taxonomic distributions (Figs.2, S3, and S4). Only taxonomic groups representing at least 1% of the total libraries are shown.

Some marked differences in proportions can be observed between the nucleic acid libraries. Groups such as the *Gemmatimonadetes* and *Deltaproteobacteria* (*Myxococcales*) highlight proportional differences between DNA and RNA groups while *Actinobacteria* were substantially higher in HRNA and *Rhizobiales* were higher in TDNA (Figs.2 and S3). Other taxonomic groups such as the *Betaproteobacteria* and *Acidobacteria* indicated a more equal representation of bacterial taxa between the four libraries (Fig.2).

**Figure 3:**
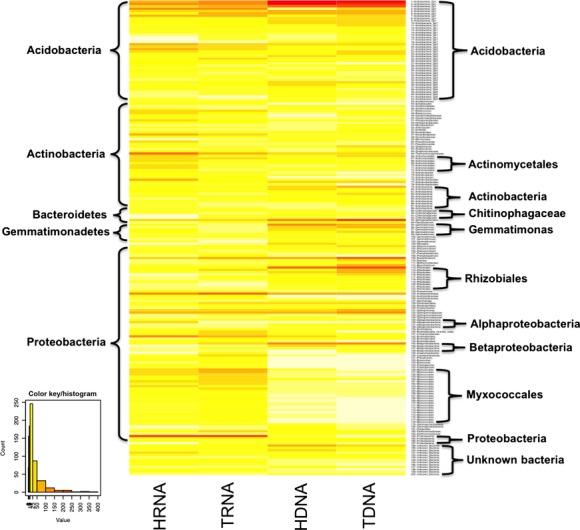
An R-generated heatmap showing the abundance distribution of the top 200 OTUs (by total number in all samples). The OTUs are sorted by taxonomic group and the samples are clustered by similarity. T(xxx) represents the total nucleic acids and H(xxx) represents the labeled nucleic acids.

While measuring sequence abundance between higher order taxonomic groups is a good indicator of overall community composition, it lacks the resolution to discern differences between individual bacteria or OTU groups. With this in mind, we constructed a heatmap of the top 200 most abundant OTUs to more closely examine individual dynamics (Fig.3). Looking at the previous higher order taxonomic heatmaps, groups such as *Acidobacteria* and *Betaproteobacteria* appeared fairly even across samples (Fig.2). This more specific taxonomic examination made differences in abundance among OTUs or groups of OTUs apparent with their absence or particularly high richness in specific samples. A number of Proteobacteria were depleted in the DNA samples, particularly the TDNA, while groups such as *Rhizobiales*, *Bradyrhizobium*, *Sphingobacteriales*, and *Acidobacteria* Gp1 showed higher abundance in one nucleic acid type over the other (DNA vs. RNA).

**Figure 4:**
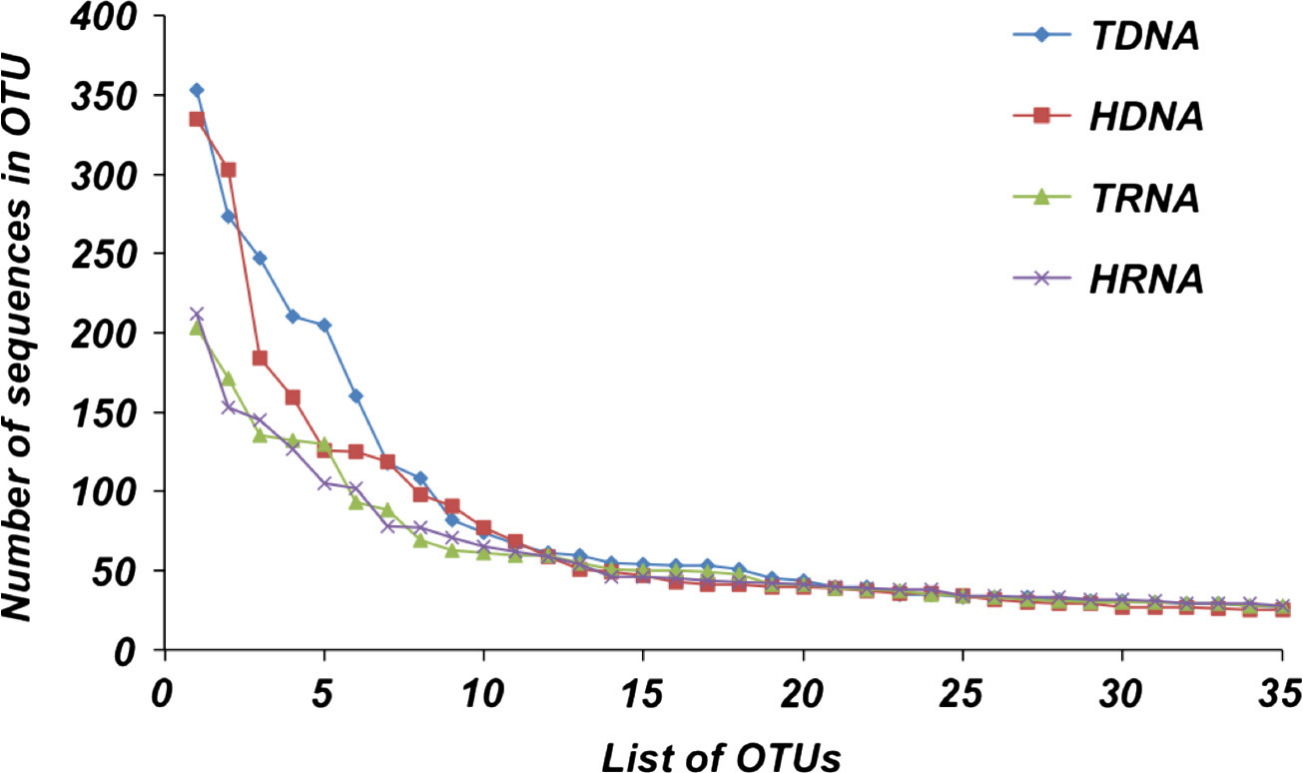
A rank-abundance plot of the four samples for the top 35 most abundant OTUs in each sample. T(xxx) represents the total nucleic acids and H(xxx) represents the labeled nucleic acids.

Some of the prominent differences between the DNA and RNA samples as well as the higher recovered richness in the RNA prompted a further investigation into the abundance distribution among the OTUs. When looking at the 10 most abundant OTU in each sample, we found that samples of the same semantide shared 80–90% of the most abundant OTUs while DNA/RNA comparisons only shared 30–40% (Table S2). Generating a rank-abundance curve revealed that the most abundant OTUs within the DNA samples had a much higher abundance than the most abundant RNA samples (Fig.4). The HDNA sample (1,617 sequences) was less ‘top heavy’ than the TDNA sample (1,830 sequences) with several of the most abundant OTUs (particularly those ranking in the top 3–6 OTUs) containing fewer total sequences, but both RNA samples (TRNA: 1,145 sequences; HRNA: 1,135 sequences) had a similar distribution across the 10 most abundant OTUs (Table S2). The abundance distribution lines of all samples converged by the 12th most abundant OTU showing a more even abundance recovery across the remaining OTUs (Fig.4).

### Total community DGGE

**Figure 5:**
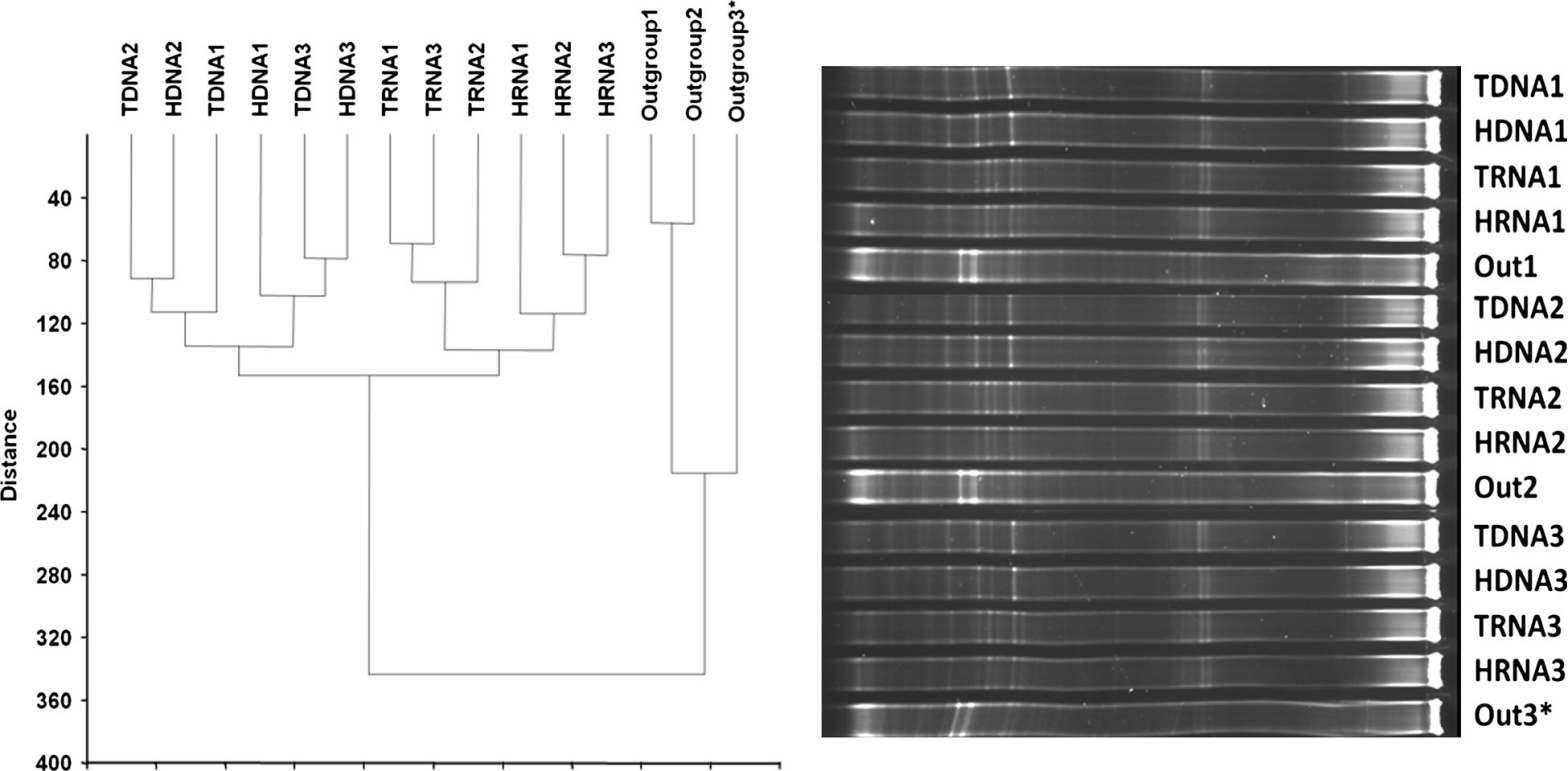
Dendogram representing DGGE community analysis profiles using the Euclidean Paired Group clustering analysis in PAST. Samples were run in triplicates. (*Indicates sample run on the end of DGGE gel with slanted bands present in profile.) T(xxx) represents the total nucleic acids and H(xxx) represents the labeled nucleic acids. Out(x) indicates the control sample which represents the microbial community from a different agricultural soil community.

In order to obtain a lower resolution analysis reflecting the soil community composition and an alternative assessment to pyrosequencing, we conducted DGGE analysis of the 16S rRNA V3-5 region. DNA from a nearby soybean field with different agronomic history was used as the out-group to root the analysis. TDNA and HDNA clustered together (Fig.5), indicating that labeled and total DNA reflected highly similar communities, while the HRNA samples clustered separately from the TRNA, indicating a separation between the two communities. This may, however, be due to proportional shifts in ribosome copy number per cell as influenced by growth rate rather than a difference in community members. Importantly, the community reflected by a DNA source was different to that reflected by RNA (Fig.5), supporting the pyrosequencing results.

## Discussion

The aim of this study was to compare how total versus newly synthesized DNA and RNA represented the soil bacterial community. Specifically we looked to characterize replicative and transcriptionally active bacterial diversity in soil using H_2_^18^O SIP and compare to total DNA and RNA extracts. The SIP libraries recovered a wide variety of taxonomic groups, suggesting that H_2_^18^O works as an effective universal label for bacterial DNA and RNA. The more dominant OTUs were represented in both labeled and total DNA and RNA extracts (Tables2 and S2). This indicated that the most abundant OTUs in total DNA and RNA extractions had active members, showing active cell division and ribosome biosynthesis. The observed differences between labeled and total nucleic acids were likely influenced by the sampling size as the OTUs unique to each sample made up a relatively small proportion of the total library. Secondly, the DNA and RNA libraries represented dissimilar overall community structures and recovered richness and diversity, highlighting the difference in community representation when comparing cell division to ribosomal synthesis.

This study avoided a common sampling bias by co-extracting both SIP-labeled and total DNA and RNA from the same soil sample and using those nucleic acid extractions for all parts of this experiment, thus removing variation between soil samples. The analyses indicated that the dominant members of the community were collected and inferences can be drawn about their recovery. However, a sampling size bias is still apparent from the rarefaction curves with the lines not approaching asymptote. The contrast between the Morisita-Horn index, shared sequence, and shared OTU data also suggests that sampling was not sufficient to describe the entire community, particularly the rare members. Although the extent of bacterial diversity in soil appears to vary by location (Roesch et al. 2007; Fulthorpe et al. 2008; Youssef and Elshahed 2009), there is evidence that extensive sequencing efforts would be necessary to sample an entire bacterial community (Gans et al. 2005; Schloss and Handelsman 2006; Roesch et al. 2007).

The growth rate of most bacteria in soil is thought to be relatively slow (Bakken 1997; Tate 2000b). However, changes in growth rate can occur with the introduction of additional resources into the system such as rhizosphere interactions (Tate 2000a) and the addition of moisture to an environment (Iovieno and Baath 2008; Blazewicz et al. 2014). Slow growth rate necessitates long incubation times for DNA-SIP in order to allow for incorporation of isotopes. If a large proportion of slow growing cells are present, only small amounts of labeled DNA may be recovered (Chen 2011). This obstacle led to the introduction of RNA-SIP as a new way to select for biologically active organisms (Manefield et al. 2002). The advantages for the use of RNA include a more rapid turnover and a better sensitivity, as RNA-SIP is independent of cell division (Manefield et al. 2002; Manefield and Whiteley 2011). The addition of water to our experimental microcosms may have helped to drive a sufficient recovery of both types of labeled nucleic acids by increasing bacterial growth (Iovieno and Baath 2008).

Our data indicated that RNA samples recovered greater richness in the soil community and the HRNA (labeled RNA) was able to recover the greatest richness. This increased recovery of richness by RNA samples can be explained by the fact that the RNA recovered sequences were not as dominated by highly abundant OTUs. The top 10 OTUs in the DNA samples made up 31–35% of all sequences while they only represented 21–22% in the RNA samples. The RNA samples were able to recover more richness because the most abundant OTUs had fewer total sequences.

Previous reports on bacterial communities compared by DNA and RNA analysis have been conflicting. While some studies have indicated that RNA and DNA recover different communities (Duineveld et al. 2001; Moeseneder et al. 2005; Dumont et al. 2011), others have suggested similarities (Nogales et al. 2001; Griffiths et al. 2003).The data from our study suggested that the semantide utilized played more of a role in the recovered richness and diversity than their indicators of biological activity (labeled nucleic acid). The Morisita-Horn analysis clearly showed the two DNA libraries and two RNA libraries were highly similar to one another (HDNA ≈ TDNA, HRNA ≈ TRNA), and the DGGE analysis supported the sequencing-based data. Both of these methods provide better resolution to dominant taxa (Muyzer and Smalla 1998; Chao et al. 2008), suggesting that the abundant populations of the labeled nucleic acid communities were very similar in proportion and diversity to the total nucleic acid communities. A recent study utilizing ^13^C-labeled substrates to study carbon cycling in soil utilizing DGGE suggested similarity in bacterial community members from labeled and unlabeled RNA (Thomson et al. 2013).

While active and total semantides of the same type were found to be similar, results indicated that DNA and RNA were disparate in both structure and overall richness and diversity. Comparing only the actively dividing bacteria (HDNA) did not substantially increase the similarity with the RNA libraries further supporting that semantide has more effect on recovery than activity. Previous studies utilizing both DNA and RNA-SIP have found that certain taxa appearing in labeled DNA are not recovered in labeled RNA (Liu and Conrad 2010; Dumont et al. 2011). These studies utilized t-RFLP fingerprinting, suggesting that the recovered taxonomic groups were dominant members of the community. We observed clear differences in abundance between RNA and DNA community members, with some taxa not being recovered for a particular nucleic acid. One of the 10 most abundant OTUs recovered from the TRNA community was not recovered at all in the TDNA sample (Table S2).

One current paradigm in soil microbial ecology is that bacteria remain dormant under nonperturbed conditions (Kell et al. 1998; Iovieno and Baath 2008; Blazewicz et al. 2013). The recovery of roughly 3X more labeled RNA than unlabeled RNA (Fig.1) in this study suggests that the majority of the bacterial community had some ribosomal activity during the 38 day incubation. We also recovered about 50% more labeled than unlabeled DNA (Fig.1) suggesting active replication took place in ∽60% of the extracted community. This may suggest that while much of the community is dormant at a particular moment, given sufficient time most soil bacteria undergo ribosomal activity and/or cell division for a least a short period of time.

Another interesting observation relates to the RNA turnover in this study. Although the majority of the RNA was labeled during the incubation period, about 25% of the recovered RNA remained unlabeled. This suggests that rRNA present at the beginning of the incubation period remained stable for over a month, despite the initial disturbance of adding water and vortexing the soil. While there is a chance that the isotope water was not readily available to all bacteria, the microcosms appeared to have been well wetted throughout. This apparent stability reinforces the idea that using rRNA as an indicator of microbial activity in soil is unreliable (Blazewicz et al. 2013). The fact that different proportions of OTUs were recovered from labeled versus the total RNA (Actinobacteria increased in HRNA), further reinforces a difference between active ribosomes and total ribosomes.

Recently synthesized (labeled) DNA and RNA represent different measures of cellular activity, and vary in number among bacteria. More importantly the copy number of amplifiable targets varies widely among taxa and growth conditions. The 16S rRNA operon number ranges among bacterial taxa from 1 to 15 copies (Pei et al. 2010) providing a potential factor of 10 difference between in representations of bacterial taxa. What we know about ribosomal copy number and growth rate is based primarily on *Escherichia coli* and other Proteobacteria. Ribosomal copy number is a factor of growth rate and varies across taxa, ranging between 6,700 and 72,000 in *E. coli* (Bremer 1996), 200-2,000 in a *Sphingomonas* sp. (Fegatella et al. 1998), and 800 to 35,000 in a *Vibrio* sp. (Flardh et al. 1992). This presents the possibility for a highly variable and disproportionate community representation depending on ribosome copy number across taxa at the time of sampling, which can vary across taxa by a factor of at least 100. This range of amplifiable targets across OTUs may explain the discrepancies of abundance in specific OTUs in DNA versus RNA extracts. As ribosomal copy number quantifications have been limited to a few bacteria in a laboratory setting, we cannot be certain of how divergent they are among bacteria in a natural soil environment. Organisms that have a metabolic advantage under particular conditions should have increased ribosome counts and therefore be reflected as increased abundance in a community, while those with a growth disadvantage would decrease; however, certain taxa can have increased ribosomal activity without cell division (Blazewicz et al. 2013). The slow growth rate and low nutrient content in soil may have caused a higher diversity recovery from RNA since the inactive portion of the RNA was smaller than in the DNA. As indicated above, about 75% of the RNA in comparison to 60% of the DNA appears to have been labeled (Fig.1). This suggests that more of the total bacterial population was represented by an increased number of ribosomes, thus presenting a greater evenness for RNA recovery. Communities in more nutrient rich environments or favorable growth conditions may not reflect the same results.

In summary, our results indicated that the majority of recovered sequences had active representatives and RNA and DNA represented dissimilar diversity. Our results also suggested that RNA more efficiently recovered the bacterial richness in the tested soil. While we acknowledge the limitations of this study due to small sampling size (duplicates of each sample) and testing of a single soil type, the focused nature of this experiment gives us some initial data on how SIP can be used to investigate total microbial diversity in a sample. This is the first known attempt to combine a concomitant SIP-labeled DNA–RNA and total DNA–RNA approach to the study of bacterial communities in an environmental sample, and is relatively unique for SIP experiments in that the labeled nucleic acids were compared against the total rather than the unlabeled nucleic acids. SIP using H_2_^18^O appeared to be effective for capturing newly synthesized nucleic acids in entire soil bacterial communities. The similarity between unlabeled and labeled nucleic acids indicated that while metabolically inactive bacteria or extracellular nucleic acids are present, most recovered OTUs had metabolically active representatives in this soil community.

## Supporting Information

Figure S1 Flowchart of procedures used during the experiment. **Figure S2.** Rarefaction curves as generated for the four individual samples. **Figure S3.** A MEGAN-generated heatmap that displays the taxonomic abundance of the samples at an order level classification. **Figure S4.** A MEGAN-generated heatmap that displays the taxonomic abundance of the samples at a family level classification.

Table S1 Roche 454 pyrosequencing primers and tags used for amplifying the V3-5 16S rRNA gene. **Table S2.** The top 10 most abundant OTUs in each sample as highlighted in red (top 1–5) and yellow (top 6–10).
