# Supplementary material for: Characterizing the diversity of active bacteria in soil by comprehensive stable isotope probing of DNA and RNA with H218O
Source: Microbiologyopen. 2015 Feb 4;4(2):208–19. doi: 10.1002/mbo3.230 (PMC4398504; doi:10.1002/mbo3.230)
Supplement: Figure S1 — Flowchart of procedures used during the experiment. Figure S2. Rarefaction curves as generated for the four individual samples. Figure S3. A MEGAN-generated heatmap that displays the taxonomic abundance of the samples at an order level classification. Figure S4. A MEGAN-generated heatmap that displays the taxonomic abundance of the samples at a family level classification. [file mbo30004-0208-sd1.docx]

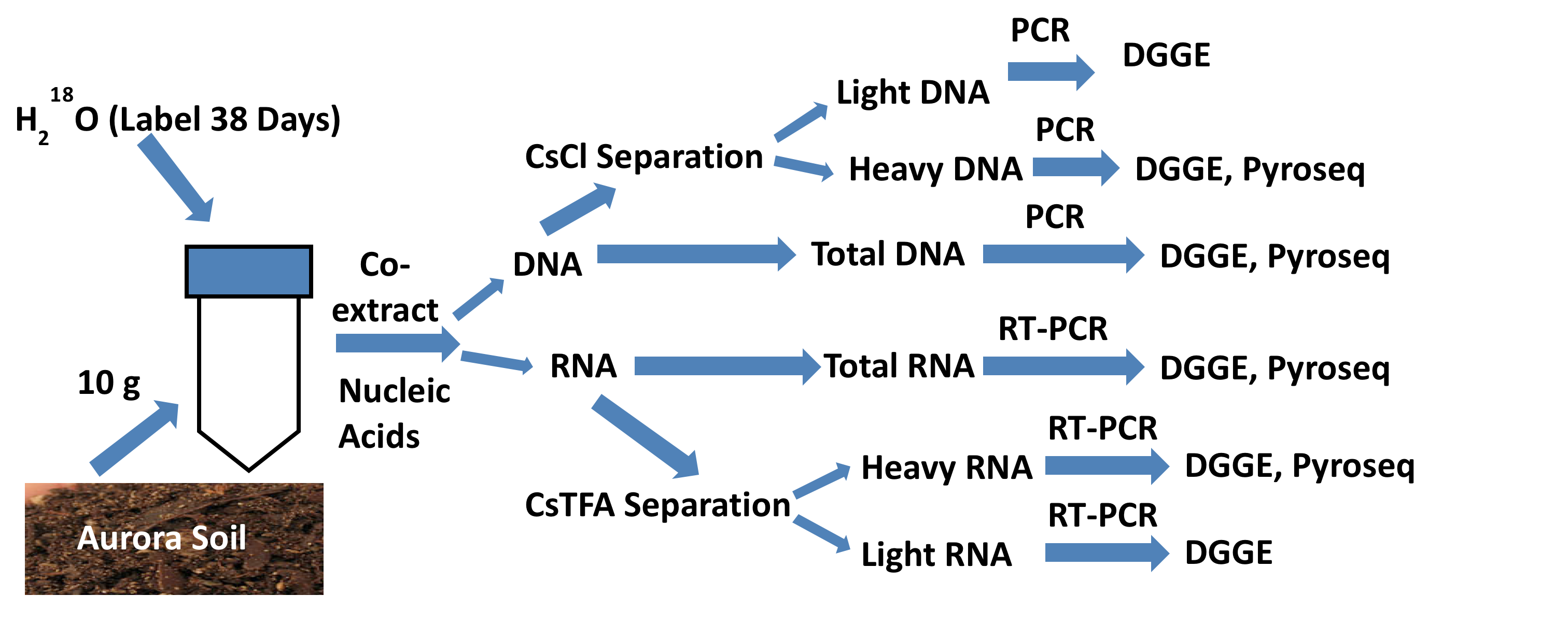


Fig. S1. Flowchart of procedures used during the experiment.


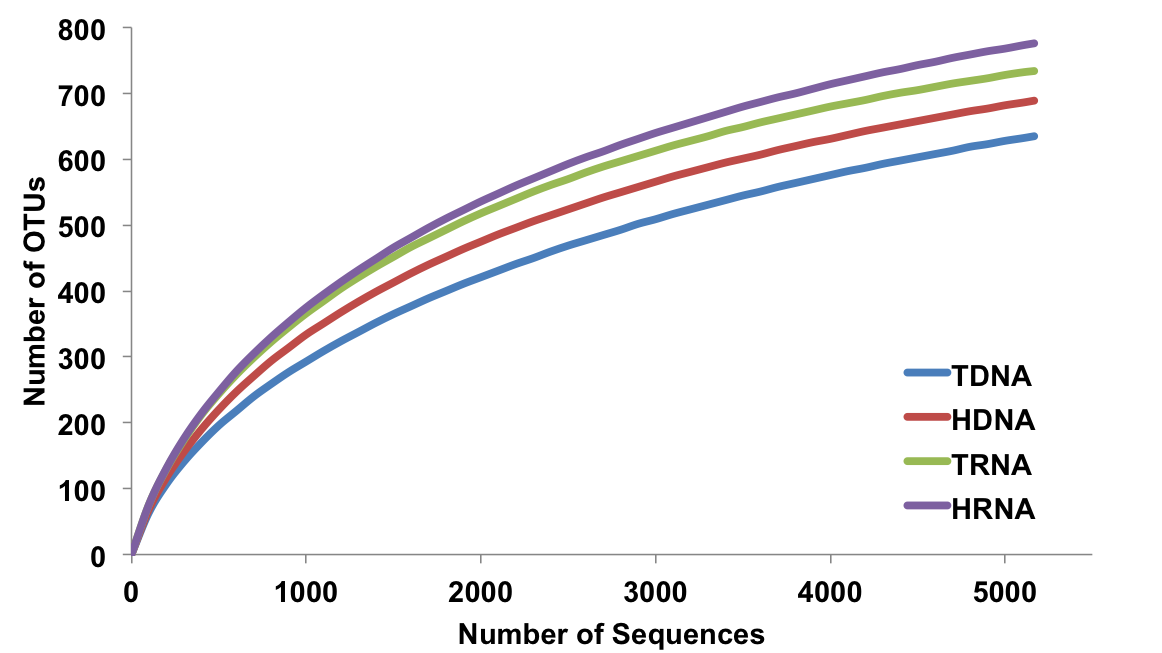


Fig. S2. Rarefaction curves as generated for the 4 individual samples.

Fig. S3. A MEGAN-generated heatmap that displays the taxonomic abundance of the samples at an order level classification.

Figure S4. A MEGAN-generated heatmap that displays the taxonomic abundance of the samples at a family level classification.

Table S1. Roche 454 pyrosequencing primers and tags used for amplifying the V3-5 16S rRNA gene.

Table S2. The top 10 most abundant OTUs in each sample as highlighted in red (top 1-5) and yellow (top 6-10).
